# Diagnostic Value of Two-Dimensional plus Four-Dimensional Ultrasonography in Fetal Craniocerebral Anomalies

**Published:** 2019-02

**Authors:** Yingjin WANG, Xiaoyuan CHEN, Shujuan ZHONG, Rong ZHANG, Yanyan PAN, Peili AN, Xinru GAO

**Affiliations:** Medical Ultrasound Centre, Northwest Women’s and Children’s Hospital, Xian 710061, P.R. China

**Keywords:** Two-dimensional ultrasound, Four-dimensional ultrasound, Fetal craniocerebral anomalies, Diagnostic value

## Abstract

**Background:**

To assess the clinical value of two-dimensional (2D) plus four-dimensional (4D) ultrasonography in diagnosis of fetal craniocerebral anomalies.

**Methods:**

Retrospective analysis was performed on the sonographic features of 83 maternity patients admitted to Northwest Women’s and Children’s Hospital, Xian China from January 2013 to December 2017 diagnosed with suspected fetal anomalies of the brain and skull through 2D and 4D ultrasonography.

**Results:**

Fifty six patients were diagnosed with the anomalies by 2D ultrasonography only, 65 patients by 4D ultrasonography only, and 74 patients by 2D plus 4D ultrasonography.76 patients were confirmed to have fetal craniocerebral anomalies after birth or induced labor. Diagnostic accuracies of 2D ultrasound only, 4D ultra-sound only, and 2D plus 4D ultrasound were 68.67%, 81.93% and 95.18%, respectively (*P*<0.05). The accuracy of 2D plus 4D ultrasound was greater than those of 2D ultrasound only and 4D ultrasound only, and the accuracy of 4D ultrasound only was higher than that of 2D ultrasound only (*P*<0.05). The sensitivity of 2D plus 4D ultrasound was greater than those of 2D ultrasound only and 4D ultrasound only (*P*<0.05). The specificity of 2D plus 4D ultrasound was greater than those of 2D ultrasound only and 4D ultrasound only (*P*<0.05).

**Conclusion:**

Combined ultrasonography can better differentiate fetal craniocerebral anomalies, providing early and more accurate information for clinicians as well as maternity patients to make a decision. This clinical practice would be valuable for improving the quality of the newborn population.

## Introduction

Fetal craniocerebral anomalies are common congenital malformations with an incidence of 5% in neonates. They are commonly described as lethal congenital malformations (1). Fetal anomalies of the brain and skull, which often occur together with a variety of non-neuronal malformations, pose formidable challenges to the society and patients’ family, in addition to negatively impacting the quality of newborn population (2). The underlying causes for fetal craniocerebral anomalies are associated with abnormalities of chromosomes and hundreds of genes. To reduce perinatal morbidity, screening for fetal craniocerebral anomalies is necessary and should be part of antepartum examination (3). However, fetal abnormalities of the brain and skull are complex and diverse due to complexity of the developing brain structure and a variety of cavities and gaps.

A single prenatal examination may not be enough to give accurate diagnosis (4). Therefore, pregnant women should undergo regular prenatal examination during the entire pregnancy to screen for fetal malformations. If any type of fetal malformation is diagnosed, a decision of whether or not to terminate the pregnancy should be made in time. Regular prenatal examination is the key to reducing the disability rate of newborns (5). Antepartum screening reduces the birth rate of malformed fetus, which can not only improve the quality of the newborn population but also promote development of the society and each family as well. It is of great diagnostic value (6).

At present, two-dimensional ultrasonography is the major and regular modality in prenatal screening due to its low cost, non-invasiveness and the lack of x-ray exposure (7). However, although 2D ultrasound is necessary and widely used in prenatal examination at all gestational weeks, it has certain limitations. Missed diagnosis or misdiagnosis of fetal craniocerebral anomalies can occur due to artifacts resulted from umbilical cord interference (8). The 4D ultrasound offers more benefits for assessing the fetus’s prenatal condition including real-time movement with much clearer images. It gives a clear look at the fetus’s general appearance and fine structures (9). Therefore, if something is suspected to be abnormal in 2D ultra-sound and a clearer picture is warranted for further assessment, a 4D ultrasound can be added to the prenatal screening (10).

In this study, retrospective analysis was performed on the sonographic features of 83 maternity patients diagnosed with suspected fetal anomalies of the brain and skull through 2D and 4D ultrasonography, aiming to explore the diagnostic effectiveness of 2D plus 4D ultrasound for fetal craniocerebral anomalies. The accuracy of 2D plus 4D ultrasound was compared with those of 2D ultrasound only and 4D ultrasound only. The findings can be used as a reference or guidance in the future use of ultrasound in prenatal screening for fetal craniocerebral anomalies.

## Materials and Methods

### Subjects

This retrospective analysis was performed on the sonographic features of 83 maternity patients admitted to Northwest Women’s and Children’s Hospital, Xian China; from January 2013 to December 2017 and were diagnosed with suspected fetal anomalies of the brain and skull through 2D and 4D ultrasound.

The study was approved by the Ethics Committee of Northwest Women’s and Children’s Hospital. The diagnostic value of 2D plus 4D ultrasound was assessed by comparison with 2D ultrasound only and 4D ultrasound only. The 83 maternity patients, aged 18–44 years, had a mean age of 27.4 ± 5.3 years. They all had a single live fetus, and were at a mean gestational age of 25.1 ± 1.8 weeks at the time of ultrasound examination. The baseline clinical records for all patients are shown in Table 1.

**Table 1:** Baseline clinical records of 83 maternity patients with suspected fetal craniocerebral anomalies, n (%)

***Variable***		***Record***
Age(yr)	<30	59 (71.08)
	≥30	24 (28.92)
Alcohol use	Yes	54 (65.06)
	No	29 (34.94)
Smoking	Yes	45 (54.22)
	No	38 (45.78)
Medication use	Yes	42 (50.60)
	No	41 (49.40)
Virus infection history	Yes	30 (36.14)
	No	53 (63.86)
Radiation exposoure	Yes	35 (42.17)
	No	48 (57.83)
Blood sugar, mmol/L	≤6.1	57 (68.67)
	>6.1	26 (31.33)
Blood routine	Hb, g/L	101±15
	RBC, 10^12^/L	3.12±1.06
	WBC, 10^9^/L	10.41±1.48
Delivery method	Vaginal	27 (32.53)
	Caesarean	56 (67.47)

### Inclusion and exclusion criteria

Patients who met the following criteria were included: 1) with suspected fetal craniocerebral anomalies diagnosed by 2D or 4D ultrasound; and 2) prenatal exposure to environmental factors that can cause fetal malformation. Patients who met the following criteria were excluded: 1) unwilling to cooperate with the examination; 2) with communication disorders or cognitive disorders; and 3) with other severe disease. All subjects and their families signed informed consent and cooperated with medical staff to complete relevant medical examination and treatment.

### Procedure for ultrasonography

Ultrasound examination was performed using a GE Voluson E8 color Doppler ultrasound system. For 2D ultrasound, a C-1-5 convex array probe for abdominal application was integrated into the ultrasound system, and the probe was set to have a frequency of 2.0–5.0 MHz. At the time of examination, the maternity patient was placed in the supine position, with the abdomen completely exposed. The abdomen was scanned with the probe moving in a multi-directional sequential and continuous way. Detailed conditions of the fetal head and neck, spine, chest and abdomen, placenta, amniotic fluid, and limbs were documented. A 4D ultrasound examination was followed after the 2D ultrasound. For 4D ultra-sound, a RIC5-9-D probe was integrated into the ultrasound system, and the probe was set to have a frequency of 3.0–7.0 MHz. The probe was moved slowly in the abdominal area in order to get clearer images. Necessary images were captured in areas with suspected fetal anomalies. A detailed sonographic analysis was performed subsequently. If a patient was diagnosed with suspected fetal craniocerebral anomalies by both 2D and 4D ultrasound, this diagnostic decision was regarded to be made from 2D plus 4D ultra-sound. A good communication was always maintained between clinicians and the maternity patient. The diagnostic accuracy, specificity and sensitivity were calculated by comparison of the sonographic findings with fetal real conditions only known after birth or induced labor.

### Types of fetal craniocerebral anomalies and prenatal sonographic features

The classification of fetal craniocerebral anomalies falls into 4 categories and 8 types. They are craniofacial deformity (encephalocele, meningocele, and anencephaly), corpus callosum deformity (corpus callosum lipoma and corpus callosum agenesis), abnormalities in head size (microcephaly and macrocephaly), and congenital hydrocephalus. Table 2 shows the relevant prenatal sonographic features.

**Table 2:** Sonographic features of 8 types of fetal craniocerebral anomalies

***Craniocerebral anomaly***	***Sonographic features***
Encephalocele	Sac/pouch containing brain tissue and cerebrospinal fluid, connected to or move with the fetal head
Meningocele	Sac/pouch containing only cerebrospinal fluid, connected to or move with the fetal head
Anencephaly	Absence of calvarium, no parenchymal tissue or only irregular masses with a mixed echo pattern above the orbits
Corpus callosum lipoma	Midline mass lesion, with associated dysgenesis of corpus callosum
Corpus callosum agenesis	Dilated lateral ventricles, absence of cavum septum pellucidum, widened space between two cerebral hemispheres, and elevated third ventricle
Microcephaly	Fetal head circumference 3 times standard deviations below the mean for gestational age and sex
Macrocephaly	Fetal head circumference 3 times standard deviations above the mean for gestational age and sex
Congenital hydrocephalus	Dilatation of lateral cerebral ventricles with ventricle/hemisphere ratio larger than 0.5

### Statistical methods

Statistical analysis was performed using SPSS 19.1 statistics software (ASIAANALYTICS FORMERLY SPSS CHINA). Count data were expressed in percentage (n (%)), and the X^2^ test was used for comparison between groups. Measurement data were expressed as X±S, and the t test was used in comparison between groups. A difference was statistically significant if *p*<0.05.

## Results

### Prenatal diagnostic results by 2D, 4D, and 2D plus 4D ultrasound and actual condition after birth or induced labor

There were no statistically significant differences in the ratio of each type of anomaly to the total anomalies detected between 2D ultrasound only, 4D ultrasound only and 2D plus 4D ultrasound (*P*>0.05). Distributions of 8 major types of fetal craniocerebral anomalies diagnosed by 2D only, 4D only and 2D plus 4D ultrasound are given in Table 3 and Table 4.

**Table 3:** Diagnostic results by ultrasound and the actual condition of the fetuses

***Craniocerebral anomaly***	***2D ultrasound only***	***4D ultrasound only***	***2D plus 4D ultrasound***	***Actual condition***
Encephalocele	2	3	4	4
Meningocele	4	6	7	7
Anencephaly	3	3	3	3
Corpus callosum lipoma	2	3	4	4
Corpus callosum agenesis	8	10	12	14
Microcephaly	6	7	8	8
Macrocephaly	10	11	12	12
Congenital hydrocephalus	21	22	24	24
Total	56	65	74	76

**Table 4:** Statistical analysis of diagnostic results by ultrasound

***Deformity categories***	***2D ultrasound only (n=56)***	***4D ultrasound only (n=65)***	***2D plus 4D ultrasound (n=74)***	***X^2^***	***P***
Craniofacial deformity	9 (16.07)	12 (18.46)	14 (18.92)	0.193	0.908
Corpus callosum deformity	10 (17.86)	13 (20.00)	16 (21.62)	0.282	0.868
Abnormalities in head size	16 (28.57)	18 (27.69)	20 (27.03)	0.038	0.981
Congenital hydrocephalus	21 (37.50)	22 (33.85)	24 (32.43)	0.374	0.829

### Diagnostic effectiveness of 2D only, 4D only and 2D plus 4D ultrasound

A total of 76 patients were confirmed to have fetal craniocerebral anomalies after giving birth or induced labor, in contrast to 56 suspected cases diagnosed by 2D ultrasound only, 65 suspected cases diagnosed by 4D ultrasound only, and 74 suspected cases diagnosed by 2D plus 4D ultrasound.

The diagnostic accuracies of the three ultrasound methods were 68.67%, 81.93%, and 95.18%, respectively.

There were statistically significant differences between the three methods (*P*<0.05). Apparently the accuracy of 2D plus 4D ultrasound was higher than those of both 2D ultrasound only and 4D ultrasound only, and the differences were statistically significant (*P*<0.01).

In addition, the accuracy of 4D ultrasound only was higher than that of 2D ultrasound only, and the difference was statistically significant (*P*<0.01). Sensitivities of the three methods were 69.74%, 82.89% and 96.05%, respectively, and the differences among them were statistically significant (*P*<0.001). The sensitivity of 2D plus 4D ultrasound was greater than those of 2D ultra-sound only and 4D ultrasound only, and the differences were statistically significant (*P*<0.001). The sensitivity of 4D ultrasound was higher than that of 2D ultrasound, but the difference was not statistically significant.

Specificities of the three methods were 57.14%, 71.43% and 85.71%, respectively, and the differences among them were statistically significant (*P*<0.01). The specificity of 2D plus 4D ultra-sound was greater than those of both 2D ultra-sound only and 4D ultrasound only, and the differences were statistically significant (*P*<0.01). The specificity of 4D ultrasound only was higher than that of 2D ultrasound only, but the difference was not statistically significant.

Diagnostic effectiveness and statistical analysis are shown in Tables 5–8.

**Table 5:** Diagnostic effectiveness of 2D ultrasound only

***Variable***		***Confirmed outcomes at end of pregnancy***		***Total***
		***Malformations***	***Normal***	
Diagnostic results by 2D ultrasound only	Malformations	53	3	56
	Normal	23	4	27
Total		76	7	83

**Table 6:** Diagnostic effectiveness of 4D ultrasound only

***Variable***		***Confirmed outcomes at end of pregnancy***		***Total***
		***Malformations***	***Normal***	
Diagnostic results by 4D ultrasound only	Malformations	63	2	65
	Normal	13	5	18
Total		76	7	83

**Table 7:** Diagnostic effectiveness of 2D plus 4D ultrasound

***Variable***		***Confirmed outcomes at end of pregnancy***		***Total***
		***Malformations***	***Normal***	
Diagnostic results by 2D plus 4D ultrasound	Malformations	73	1	74
	Normal	3	6	9
Total		76	7	83

**Table 8:** Statistical analysis of diagnostic effectiveness by three ultrasound methods

***Performance***	***2D ultrasound only***	***4D ultrasound only***	***2D plus 4D ultrasound***	***X^2^***	***P***
Accuracy, %	68.67	81.93^*^	95.18^*#^	19.690	<0.001
Sensitivity, %	69.74	82.89	96.05^*#^	18.560	<0.001
Specificity, %	57.14	71.43	85.71^*#^	3.616	0.042

* Compared with 2D ultrasound only, *P*<0.05;

# Compared with 4D ultrasound only, *P*<0.05

## Discussion

Although sometimes it is a genetic problem, in most cases, exposure to environmental factors such as certain medicines, infections or radiation during pregnancy contributes to fetal craniocerebral anomalies. Misuse of drugs like progestogens, estrogens and androgens that pregnant women often use in early pregnancy may potentially cause fetal malformations of the brain and skull. Therefore, precautions should be taken during pregnancy when taking a medication, and the best way is seeking guidance from a physician. Tetracycline and streptomycin were all proved to result in fetal craniocerebral anomalies if being administered for a long time by pregnant women. Excessive exposure to radiation can lead to fetal anomalies of the brain and skull as well, and even with the risk of stillbirth, congenital heart disease, etc. Infections of pregnant women with virus such as influenza virus, cytomegalovirus, herpes simplex virus, rubella virus, etc. can lead to fetal microcephaly, brain calcification, and hydrocephalus, and sometimes combined with other organ malformations. Long-term consumption of tobacco and alcohol abuse by to-be parents can cause fetal anomalies of the brain and skull, stillbirths, and fetal mental retardation (11, 12). The underlying mechanism of fetal malformations of the brain and skull is genetic mutations, which may be induced by medications, radiation and diseases etc. during pregnancy (13).

At present, 2D ultrasound is the routine modality for prenatal screening of fetal craniocerebral anomalies. It becomes popular and widely used in prenatal examination of pregnant women due to its non-invasiveness, low cost, and the lack of xray exposure (14). However, 2D ultrasound can only give the cross-sectional images of a certain part of the fetus, and it can neither display fetal subtle structural features, nor provide a clearer and more effective stereo image. Thus, 2D ultra-sound has certain limitations such as artifacts and low resolution, which compromise the diagnostic accuracy (15). As modern medical technologies advance, medical instruments become more sophisticated. In this trend, 4D ultrasound has become an important supplement to 2D ultrasound (16). 4D ultrasound can scan the target from multiple angles giving real-time multi-layer images. Thus, the fetus’s subtle structure, including the overall morphology, structures and the spatial relationships of each body part, can be observed directly and clearly (17). Using 4D ultrasound in prenatal screening, fetal intracranial cysts can be better visualized for more accurate differentiated diagnosis, by observing the focal location, size and structure, the spatial relationship with surrounding brain parenchyma, as well as the image stability when the fetus moves in different direction (18). In general, 4D ultrasound performs better in prenatal screening of the fetus, and its clinical application should be promoted.

In this study, 56 of 83 enrolled patients were diagnosed with suspected brain malformations by 2D ultrasound only, 65 by 4D ultrasound only, and 74 by 2D plus 4D ultrasound. There were no statistically significant differences in the ratio of each type of malformation to the total malformations detected among 2D ultrasound only, 4D ultrasound only and 2D plus 4D ultrasound. The findings in this study were consistent with a report published by Snoek et al. (19). At the end of pregnancy, 76 patients were confirmed to have fetal craniocerebral anomalies after giving birth or induced labor. Diagnostic accuracies of 2D ultrasound only, 4D ultrasound only, and 2D plus 4D ultrasound were 68.67%, 81.93% and 95.18%, respectively, and the differences among them were statistically significant. The accuracy of 2D plus 4D ultrasound was greater than those of 2D ultrasound only and 4D ultrasound only, and the accuracy of 4D ultrasound only was higher than that of 2D ultrasound only. The differences above were all statistically significant. Sensitivities of the three groups were 69.74%, 82.89% and 96.05%, respectively, and the differences among them were statistically significant. The sensitivity of 2D plus 4D ultrasound was greater than those of 2D ultrasound only and 4D ultrasound only, and the differences were statistically significant. The sensitivity of 4D ultrasound only was higher than that of 2D ultrasound only, but the difference was not statistically significant. Specificities of the three groups were 57.14%, 71.43% and 85.71%, respectively, and the differences among them were statistically significant. The specificity of 2D plus 4D ultrasound was greater than those of 2D ultrasound only and 4D ultrasound only, and the differences were statistically significant. The specificity of 4D ultrasound only was higher than that of 2D ultrasound only, but the difference was not statistically significant. The findings in this study were consistent with a report in literature (20), and suggested that 2D plus 4D ultrasound can differentially diagnose various types of fetal craniocerebral anomalies more accurately than 2D ultrasound only and 4D ultrasound only.

The sample size in this study was small due to limited number of patients with fetal craniocerebral anomalies admitted to Northwest Women’s and Children’s Hospital, thus the results may contain some chance findings leading to a nonnegligible small sample bias. A longer follow-up was planned for the subjects in this study. In our future studies, improvement will be made in the study design aimed at achieving the best study results.

## Conclusion

2D plus 4D ultrasound can better differentiate various fetal craniocerebral anomalies, providing early and more accurate information for clinicians as well as maternity patients to make a decision. This clinical practice would be valuable for improving the quality of the newborn population.

## Ethical considerations

Ethical issues (Including plagiarism, informed consent, misconduct, data fabrication and/or falsification, double publication and/or submission, redundancy, etc.) have been completely observed by the authors.
